# Urban-rural inequities in knowledge, attitudes and practices regarding tuberculosis in two districts of Pakistan's Punjab province

**DOI:** 10.1186/1475-9276-10-8

**Published:** 2011-02-04

**Authors:** Muhammad Umair Mushtaq, Ubeera Shahid, Hussain Muhammad Abdullah, Anum Saeed, Fatima Omer, Mushtaq Ahmad Shad, Arif Mahmood Siddiqui, Javed Akram

**Affiliations:** 1 Allama Iqbal Medical College, Lahore, Pakistan; 2 Department of Health, Government of Punjab, Lahore, Pakistan

## Abstract

**Objective:**

The aim of this study was to explore inequities in knowledge, attitudes and practices regarding tuberculosis (TB) among the urban and rural populations.

**Design:**

A cross-sectional study was conducted in two districts of Pakistan's Punjab province. The 1080 subjects aged 20 years and above, including 432 urban and 648 rural respondents, were randomly selected using multistage cluster sampling and interviewed after taking verbal informed consent. Logistic regression was used to calculate the crude odds ratio (OR) with 95% confidence interval (CI) for the urban area. The differences in knowledge, attitudes, practices and information sources between the urban and rural respondents were highlighted using Pearson chi-square test and Fisher's exact test.

**Results:**

The study revealed poor knowledge regarding TB. The deficit was greater in the rural areas in all aspects. The knowledge regarding symptoms (OR 2.03, 95% CI 1.59-2.61), transmission (OR 1.93, 95% CI 1.44-2.59), prevention (OR 2.24, 95% CI 1.70-2.96), duration of standard treatment (OR 1.88, 95% 1.41-2.49) and DOTS (OR 1.84, 95% CI 1.43-2.38) was significantly higher in the urban areas (all P < 0.001). Although a majority of the subjects (urban 83.8%, rural 81.2%) were aware of the correct treatment for TB, less than half (urban 48.1%, rural 49.2%) were aware of the availability of the diagnostic facility and treatment free of cost. The practice of seeking treatment at a health facility (P = 0.030; OR 2.01, 95% CI 1.06-3.82), as soon as they realized that they had TB symptoms (P < 0.001; OR 1.72, 95% CI 1.26-2.35), was significantly higher in the urban areas. People in the urban areas were more likely to feel ashamed and embarrassed being a TB patient (P < 0.001; OR 2.03, 95% CI 1.50-2.76); however, they seem to be supportive in case their family member suffered from TB (P = 0.005; OR 1.53, 95% CI 1.13-2.06). Nearly half of the respondents, irrespective of the area of residence, believed that the community rejects the TB patient (urban 49.8%, rural 46.4%). Television (urban 80.1%, rural 68.1%) and health workers (urban 30.6%, rural 41.4%) were the main sources for people to acquire the TB related information.

**Conclusion:**

Respondents' knowledge regarding TB was deficient in all aspects, particularly in the rural areas. Intended health seeking behavior was better in the urban areas. Television and health workers were the main sources for TB related information in both the urban as well as the rural areas. Therefore, the area of residence should be considered in tailoring communication strategies and designing future interventions for TB prevention and control.

## Introduction

Each year about 9 million people become victims of tuberculosis (TB) and 1.6 million die worldwide [1]. A World Health Assembly resolution invigorated the TB control efforts in 1991 and the internationally recommended control therapy, later named DOTS, was launched in 1994 [2,3]. Globally, Pakistan ranks eighth for the high TB incidence. In Pakistan, the prevalence of TB is 297 cases per 100,000 population and nearly 0.3 million new cases arise each year [1]. Pakistan adopted DOTS in 1995 and TB was declared as a national emergency in 2001 [4]. Pakistan's TB control program failed to meet the World Health Organization's 2005 target of 70% case detection and 85% cure, and is not yet on track to meet the Millennium Development Goals by 2015 [1]. Lack of knowledge about the disease and stigmatization causes underutilization of the services, delay in seeking diagnosis, and poor treatment compliance [5,6].

Several international studies have reported poor knowledge, attitudes and practices about TB [7-30]. Many studies in Pakistan revealed poor TB awareness and stigmatization, however, only a few of these studies were community-based [31-36]. In Pakistan, nearly 65% of the population resides in the rural areas [37], yet no study has explored the differences in knowledge, attitudes and practices regarding TB between the urban and rural populations. The province of Punjab accounts for over 50% of Pakistan's TB burden with 177 cases occurring per 100,000 people [38]. Punjab has a population of about 90 million and nearly 70% of this population resides in the rural areas [37]. This paper provides the baseline assessment of knowledge, attitudes and practices regarding TB in an urban-rural perspective, in two districts of the Punjab province of Pakistan, to help in drafting better strategies for advocacy, communication and social mobilization.

## Methods

### Design, Settings and Sample

A cross-sectional study was conducted in 2008-09. Out of the 35 districts in Punjab, two districts were selected at random, namely Nankana Sahib from central Punjab and Bahawalnagar from southern Punjab, having a combined population of 4.6 million. [see Additional file 1]

By multi-stage cluster sampling, three *tehsils *(sub districts) were selected randomly from each district. Next, five union councils, two urban and three rural (according to the urban-rural population proportion in Pakistan) were picked randomly from each *tehsil*. Two villages (in case of the rural union councils) or two electoral wards (in case of the urban union councils) were randomly selected from each union council and eighteen individuals were interviewed from each village/electoral ward (18 respondents × 2 villages/electoral wards × 5 union councils × 3 *tehsils *× 2 districts = 1080 respondents). [see Additional file 2]

Selection of the direction of first household was done as follows: the interviewers moved to the center of the village/electoral ward, spun a bottle and continued in the direction where the bottle pointed. Respondents aged 20 years and above were included in the study after taking their verbal informed consent. Health care providers and respondents with a prior history of TB or those receiving TB treatment were excluded.

Sample size was calculated by using Epi Info 6.04d (2004) [39]. With the expected frequency of awareness of 12% [15], confidence interval of 95%, margin of error of ± 2 and 5% design effect, a sample size of 1064 was deemed sufficient and it was rounded off to 1080.

### Data Collection

A semi-structured questionnaire was designed according to the World Health Organization guidelines [40], and translated into Urdu (National language of Pakistan). The questionnaire consisted of sociodemographic questions (area, occupation, education, family members, family income and housing condition), knowledge about TB (symptoms, transmission, prevention, treatment, and seriousness of disease), attitudes and practices (intended health seeking behavior, consultation about the disease, relation of family and community members with the patients and patient's quality of life) and information sources. [see Additional file 3].

The questionnaire was pre-tested in the field and modified accordingly. Medical students trained in the interviewing techniques filled the questionnaire during household visits and a field coordinator monitored the entire process. Health education was also provided as a part of the study.

The study was approved by the Ethical Review Board of Allama Iqbal Medical College, Lahore, Pakistan. Due to non-invasive nature of the study, verbal informed consent was deemed sufficient and an informed consent form was included in the questionnaire.

### Data Analysis

The data was analyzed by using SPSS version 17.0 (SPSS Inc. Chicago, Illinois, 2008). Descriptive statistics were used to evaluate the urban-rural differences in knowledge, attitudes and practices regarding TB. Logistic regression was used to calculate crude odds ratio (OR) with 95% confidence interval (CI) for the urban areas. Pearson chi-square test and Fisher's exact test were used to compare data among the urban and rural areas. All tests were 2-sided and the statistical significance was considered at *P *< 0.05.

## Results

Of the 1080 respondents, 432 (40%) were from the urban areas and 648 (60%) were from the rural areas. Among 432 respondents from the urban areas, 76.2% were male and 23.6% were female while among 648 respondents from the rural areas, 76.7% were male and 23.3% were female. Most of the respondents (urban 66.4%, rural 58.5%) were 31-50 years of age and the rest were either less than 30 years (urban 20.1%, rural 20.5%) or above 50 years (urban 13.4%, rural 21.0%). The 56.3% urban and 51.9% rural respondents had the high school education, 27.1% urban and 22.7% rural respondents had the primary school education, and 16.3% urban and 25.5% rural respondents were illiterate. Mean persons per household were 6.70 ± 2.63 in the urban areas and 6.81 ± 3.27 in the rural areas while mean number of working members per household were 1.92 ± 1.12 in the urban areas and 1.95 ± 1.27 in the rural areas. Majority of the population (urban 41.2%, rural 59.3%) had low per capita income, followed by middle (urban 39.2%, rural 31.8%) and high (urban 19.6%, rural 8.9%) per capita income.

### Knowledge

#### Symptoms

Cough was the most commonly stated symptom in both the urban (66.2%) and rural (63.4%) areas. Other symptoms cited were: coughing up blood (urban 56.5%, rural 50.0%), weight loss (urban 57.4%, rural 38.0%), fever (urban 45.4%, rural 51.7%), fever >7 days (urban 24.5%, rural 13.9%), chest pain (urban 30.8%, rural 21.0%), shortness of breath (urban 37.7%, rural 28.7%), fatigue (urban 40.3%, rural 32.7%), severe headache (urban 12.7%, rural 8.6%) and nausea (urban 21.1%, rural 14.0%).

'Cough lasting longer than three weeks' was considered the correct statement regarding knowledge about the symptoms of TB and that was cited by 53.5% urban and 36.1% rural respondents (P < 0.001; OR 2.03, 95% CI 1.59-2.61). [Table 1].

**Table 1:** Knowledge regarding tuberculosis among respondents in the rural and urban areas of two districts of Pakistan's Punjab province (n = 1080)

Knowledge	Urban (n = 432)	Rural (n = 648)	P value	OR (95% CI)
			
	n (%)	n (%)		
**Symptoms**				
*Cough that lasts longer than 3 weeks*	231 (53.5)	234 (36.1)	P < 0.001	2.03 (1.59-2.61)
Coughing up blood	244 (56.5)	324 (50.0)	P = 0.037	1.30 (1.02-1.66)
Weight loss	248 (57.4)	246 (38.0)	P < 0.001	2.20 (1.72-2.82)
Fever	196 (45.4)	335 (51.7)	P = 0.042	0.78 (0.61-0.99)
**Transmission**				
*Through the air when a patient coughs or sneezes*	349 (80.8)	444 (68.5)	P < 0.001	1.93 (1.44-2.59)
**Prevention**				
*Covering mouth and nose while coughing or sneezing*	338 (78.2)	399 (61.6)	P < 0.001	2.24 (1.70-2.96)
By drugs (chemoprophylaxis/treatment of patient)	193 (44.7)	267 (41.2)	P = 0.258	1.15 (0.90-1.47)
By vaccination (BCG)	189 (43.8)	212 (32.7)	P < 0.001	1.60 (1.24-2.06)
**Treatment**				
Mode of treatment - *specific drugs given by health center*	362 (83.8)	526 (81.2)	P = 0.269	1.20 (0.87-1.66)
Place of treatment - *government hospital or private clinic*	427 (98.8)	629 (97.1)	P = 0.053	2.58 (0.96-6.96)
Duration of treatment - *6 to 9 months*	299 (69.2)	356 (54.9)	P < 0.001	1.84 (1.43-2.38)
Cost of treatment - *free of charge*	208 (48.1)	319 (49.2)	P = 0.728	0.96 (0.75-1.22)
Knowledge about DOTS	130 (30.1)	121 (18.7)	P < 0.001	1.88 (1.41-2.49)

***Note: ***The responses considered correct are in '*italics*'

#### Transmission and prevention

Possible modes of transmission indicated by the respondents were sharing food utensils (urban 35.6%, rural 29.6%), eating in the same plate (urban 44.7%, rural 44.1%), shaking hands (urban 3.0%, rural 5.4%) and touching items in the public places (urban 0.0%, rural 1.7%). When inquired about the preventive measures for TB, responses included: avoid sharing food utensils (urban 44.7%, rural 34.6%), avoid shaking hands (urban 5.1%, rural 8.8%), washing hands after touching items in the public places (urban 3.7%, rural 12.3%), maintaining good nutrition (urban 36.8%, rural 31.5%), drugs (urban 44.7%, rural 41.2%) and BCG vaccination (urban 43.8%, rural 32.7%). Praying (urban 19.9%, rural 19.4%) and closing windows at home (urban 1.6%, rural 0.6%) were also mentioned.

'Through the air when a TB patient coughs or sneezes (droplet infection)' was considered the correct statement regarding knowledge about the transmission of TB and this statement was cited by 80.8% urban and 68.5% rural respondents (P < 0.001; OR 1.93, 95% CI 1.44-2.59). 'Covering the mouth and nose while coughing and sneezing' was considered the correct statement regarding knowledge about the prevention of TB and this statement was responded by 78.2% urban and 61.6% rural interviewees (P < 0.001; OR 2.24, 95% CI 1.70-2.96). [Table 1].

#### Treatment

Respondents were asked about the treatment options according to the National TB Control Program (NTP) guidelines. Regarding the mode of treatment, responses included specific drugs given by the health centers (urban 83.8%, rural 81.2%), DOTS (urban 30.1%, rural 18.7%), praying (urban 19.4%, rural 17.7%), herbal remedies (urban 0.9%, rural 4.8%) and home rest (urban 1.2%, rural 1.7%). Place of treatment was responded as government hospitals and/or private clinics by majority of the people (urban 98.7%, rural 97.1%). Other responses included traditional/homeopathic healers (urban 1.6%, rural 3.9%) and non-governmental organization clinics (urban 1.2%, rural 1.5%). Duration of the treatment of TB as reported by the respondents was 6-9 months (urban 69.2%, rural 54.9%), greater than 9 months (urban 19.4%, rural 22.1%), 3-6 months (urban 6.9%, rural 13.4%) and less than 3 months (urban 4.4%, rural 9.6%). The 48.1% urban and 49.2% rural respondents indicated that the treatment is free of cost while others believed it to be reasonably priced (urban 21.5%, rural 19.9%), expensive (urban 11.5%, rural 15.4%) and very expensive (urban 18.5%, rural 15.4%).

Correct knowledge about the mode, place and cost of TB treatment was not significantly associated with the area of residence, however, a correct response regarding the duration of treatment (6-9 months) was significantly higher in the urban areas (P < 0.001; OR 1.88, 95% CI 1.41-2.49). Knowledge about DOTS was also higher in the urban areas (P < 0.001; OR 1.84, 95% CI 1.43-2.38). [Table 1].

### Attitudes and practices

#### Intended health seeking behavior

When asked what they would do if they had symptoms of TB, 97.0% urban and 94.1% rural respondents answered that they would visit a health facility. Other responses included visiting a nearby pharmacist (urban 2.1%, rural 4.5%), going to the traditional healers (urban 0.5%, rural 3.5%) and initiating self-treatment (urban 0.2%, rural 1.4%). Most of the respondents said they would seek treatment as soon as they realized that they had symptoms of TB (urban 83.6%, rural 74.7%). Others said they would go to a health facility if symptoms lasted for 3-4 weeks (urban 14.6%, rural 18.5%) or self-treatment failed (urban 1.6%, rural 5.2%) while some would not go to a doctor (urban 0.2%, rural 1.5%).

The practice of seeking treatment at a health facility (P = 0.030; OR 2.01, 95% CI 1.06-3.82) as soon as they realized that they had symptoms of TB (P = 0.001; OR 1.72, 95% CI 1.26-2.35) was significantly higher in the urban areas. [Table 2].

**Table 2:** Attitudes and practices regarding tuberculosis among respondents in the rural and urban areas of two districts of Pakistan's Punjab province (n = 1080)

Attitude and Practices	Urban (n = 432)	Rural (n = 648)	P value	OR (95% CI)
			
	n (%)	n (%)		
**What would you do if you suspect you had symptoms of TB? (*Intended health seeking behavior)***				
Health Facility	419 (97.0)	610 (94.1)	P = 0.030	2.01 (1.06-3.82)
Pharmacy	9 (2.1)	29 (4.5)	P = 0.037	0.45 (0.21-0.97)
Traditional Healer	2 (0.5)	23 (3.5)	P = 0.001	0.13 (0.03-0.54)
Self Treatment	1 (0.2)	9 (1.4)	P = 0.058	0.17 (0.02-1.31)
Others	6 (1.4)	8 (1.2)	P = 0.826	1.13 (0.39-3.27)
**When you will go to a health facility after getting TB? (*Intended health seeking behavior)***				
When treatment on my own does not work	7 (1.6)	34 (5.2)	P = 0.002	0.30 (0.13-0.68)
When symptoms last for 3 to 4 weeks	63 (14.6)	120 (18.5)	P = 0.091	0.75 (0.54-1.05)
As soon as I realize that my symptoms are of TB	361 (83.6)	484 (74.7)	P = 0.001	1.72 (1.26-2.35)
I would not go to Doctor	1 (0.2)	10 (1.5)	P = 0.058	0.15 (0.02-1.16)
**How a TB patient is regarded in your community? *(Community stigma)***				
Most people reject	215 (49.8)	302 (46.6)	P = 0.308	1.14 (0.89-1.45)
Most people friendly but avoid	138 (31.9)	210 (32.4)	P = 0.873	0.98 (0.75-1.27)
Most people support and help	79 (18.3)	136 (21.0)	P = 0.276	0.84 (0.62-1.15)
**What would be your reaction if you were found out that you have TB? *(Personal stigma)***				
Shame and/or embarrassment	361 (83.6)	463 (71.5)	P < 0.001	2.03 (1.50-2.76)
Fear and/or hopelessness	151 (35.0)	237 (36.6)	P = 0.587	0.93 (0.73-1.20)
Others	28 (6.5)	102 (15.7)	P < 0.001	0.37 (0.24-0.58)
**How your relationship would changes if your close family member gets TB? *(Family support to the patient)***				
Sympathy, support and help	352 (81.5)	481 (74.2)	P = 0.005	1.53 (1.13-2.06)
Hate	6 (1.4)	28 (4.3)	P = 0.007	0.31 (0.13-0.76)
Friendly but avoid	74 (17.1)	139 (21.5)	P = 0.081	0.76 (0.55-1.04)

#### Stigma

Most of the respondents believed that the community (urban 49.8%, rural 46.6%) rejects the TB patient. Others believed that people are friendly but they avoid the patient (urban 31.9%, rural 32.4%) or support the patient (urban 18.3%, rural 21.0%). When asked what their reaction would be if they themselves had TB, majority of the people responded they would experience shame and/or embarrassment (urban 83.6%, rural 71.5%) while others expressed fear and/or hopelessness (urban 35.0%, rural 36.6%). If a close family member gets TB, respondents would hate the patient (urban 1.4%, rural 4.3%), would be friendly but avoid the patient (urban 17.1%, rural 21.5%) or would be sympathetic and supportive to the patient (urban 81.5%, rural 74.2%).

Those living in the urban areas were more likely to feel ashamed of and embarrassed being a TB patient (P < 0.001; OR 2.03, 95% CI 1.50-2.76); however, they would be supportive if their family member suffered from TB (P = 0.005; OR 1.53, 95% CI 1.13-2.06). The community would reject the TB patient irrespective of the area of residence (P = 0.470). The quality of life of the patients was perceived as normal (urban 64.8%, rural 50.5%), poor (urban 25.5%, rural 37.2%) and very poor (urban 9.7%, rural 12.0%) while a few (urban 0.0%, rural 0.3%) responded it to be good. [Table 2].

### Information sources

Main information sources were television (urban 80.1%, rural 68.1%) and health workers (urban 30.6%, rural 41.4%). Other sources are listed in Table 3. Most of the people watch television after 6 pm (urban 75.2%, rural 68.4%) and the most viewed television channels were Pakistan Television (urban 65.0%, rural 65.7%) and Geo Television (urban 22.5%, rural 2.8%).

**Table 3:** Sources of information regarding tuberculosis among respondents in the rural and urban areas of two districts of Pakistan's Punjab province (n = 1080)

Sources of information	Urban (n = 432)	Rural (n = 648)	P value	OR (95% CI)
			
	n (%)	n (%)		
Television	346 (80.1)	441 (68.1)	P < 0.001	1.89 (1.42-2.52)
Radio	49 (11.3)	61 (9.4)	P = 0.305	1.23 (0.83-1.83)
Newspapers and magazines	60 (13.9)	62 (9.6)	P = 0.028	1.52 (1.05-2.23)
Banners and paintings	1 (0.2)	14 (2.2)	P = 0.008	0.11 (0.01-0.80)
Brochures, posters and other printed materials	29 (6.7)	56 (8.6)	P = 0.249	0.76 (0.48-1.21)
Health workers	132 (30.6)	268 (41.4)	P < 0.001	0.62 (0.48-0.81)
Family, friends, neighbors and colleagues	27 (6.3)	27 (4.2)	P = 0.124	1.53 (0.89-2.65)
Religious leaders	1 (0.2)	17 (2.6)	P = 0.003	0.09 (0.01-0.65)
Teachers	10 (2.3)	49 (7.6)	P < 0.001	0.29 (0.15-0.58)
Other	3 (0.7)	5 (0.8)	P = 1.000	0.89 (0.21-3.78)

## Discussion

Pakistan has a vertical TB control system under the National TB Control Program (NTP). DOTS coverage is 100% and free diagnostic and treatment facilities are available at all government hospitals and health centers. However, Pakistan still ranks eighth globally for the high TB incidence.

Previous studies revealed that the treatment facilities available at health centers especially in the rural areas are limited and ineffective [1,41,42], and there are inherent problems with the system of patient referral to the DOTS facility. The private sector is not involved in DOTS implementation and most people first report to a private practitioner for their symptoms [43]. Aside from the rare cases of self-referrals, accessibility to DOTS relies primarily on referral by the private practitioners or government health workers working in primary health centers. However, many of these private practitioners and government health workers are poorly trained in the diagnosis and treatment of TB and lack the communication skills required to motivate the patient for increased compliance [44,45]. The situation is further aggravated by a poor knowledge about the disease among patients. Therefore, referral to the facility providing DOTS does not work well in most of the cases [7,46].

In accordance with previous studies in Pakistan [31-34], the knowledge of the disease was poor especially in the rural areas. Majority of the respondents answered cough as the presenting complaint, in consistence with a Chinese study [7], most were not aware that an episode of cough lasting longer than 3 weeks was an alarm sign for TB. This finding has an important implication for the TB control program in Pakistan because the knowledge regarding when to report a health facility is essential for the success of a program based on passive case finding. The respondents had the basic knowledge about transmission, prevention and treatment of TB, consistent with the studies elsewhere [10-13,32]. The awareness again showed an increasing trend in the urban areas that necessitates the need to focus on the rural areas. Most people were unaware of BCG vaccination as a preventive measure in children despite 90% BCG coverage in Pakistan [47], in agreement with a study in Sind province of Pakistan [33]. Knowledge about the duration of standard treatment was limited consistent with the previous studies [31,32]. More grave was the fact that less than half of the people knew about the availability of free diagnosis and treatment facilities. Limited knowledge regarding free diagnosis and treatment has been reported previously in Pakistan and China [7,35]. Since Pakistan's TB control program relies on self-referral, this finding holds special importance from a TB control perspective. If the people are unaware of free diagnostic and treatment facilities, they are less likely to show up for diagnostic evaluation and treatment. Poor awareness especially regarding symptoms and treatment results in delayed case finding and poor treatment adherence [8,12,48-51], both of which are a problem in Pakistan [1,6].

Lack of awareness has been associated with the health-seeking behaviors [14,16]. Many people do not seek early diagnosis and treatment especially in the rural areas because they may not suspect TB upon appearance of early symptoms (cough, fever, etc.) unless severe symptoms (hemoptysis, weight loss, etc.) set in. The health-seeking behavior was better in the urban communities; the reasons for this could possibly be ease in access to the health facilities and better knowledge about the disease [15]. Most respondents would go to a health facility as soon as they realize they had symptoms of TB in agreement with the previous studies [12,14,52], while 25% rural and 17% urban respondents would delay seeking treatment. Some respondents believed in prayer as a treatment option. The reason for this could lie in the strong belief in spiritual healers or 'pirs' in Pakistani society. Stigmatization associated with TB might be another major reason for delayed diagnosis and poor treatment compliance.

Majority of the respondents, especially those living in the urban areas, indicated that they would feel embarrassed and ashamed if they found they have TB. Similar feelings have long been associated with TB [10,17-19,22-25,53]. Personal stigma results from a fear of transmitting infection and avoiding potential discrimination from the community [20]. Nearly half of the respondents, irrespective of the area of residence, believed that the community rejects them TB patient. Community stigma stems from a perceived risk of infection and perceived link between TB and poverty, low caste, disreputable behavior and divine punishment [20]. It can be further explained by the values deep rooted in the cultural fabric of South Asia, where a TB patient has long been condemned, disgraced and marginalized by the society [31-33,21]. Social stigma adversely affects the sufferers and the impact is felt at home, in the workplace and the community. It results in delay in seeking treatment and poor treatment compliance [10,26,27,54]. The study revealed that most of the people, especially in the urban areas, would be supportive if their family member suffered TB. The rural respondents were more likely to hate a TB patient in the family. Stigmatization was inversely associated with the degree of kinship in previous studies [10,12,28,29]. Moral support provided by the families often plays a vital role in the early diagnosis and treatment compliance [10,35,22,55]. Increasing TB awareness can reduce discrimination and stigma as the lack of basic knowledge about the disease is an important contributing factor in stigmatization associated with TB [16,20,22,56].

Lack of awareness is an important risk factor for exposure to TB and it not only affects health-seeking behaviors but also the control strategy, thereby sustaining transmission of disease within the population [8,9,48-50,57-59]. The low level of knowledge about TB among people, especially those living in the rural areas, was revealed in the current study. It reflects the ineffective communication strategy of Pakistan's NTP to reach all the people despite the fact that two-third of Pakistan's population resides in the rural area. Pakistan's NTP designed the communication strategy for 2005-10 but it still awaits implementation [4]. Information, education and communication (IEC) regarding TB is provided by the primary health centers and their out-reach staff. These community health workers have proved to be the most cost-effective in DOTS implementation in Pakistan [60]. Although DOTS population coverage is almost 100%, the lack of effective communication skills among the health workers is the main constraint. In Bangladesh, well-conducted community education campaigns have shown to produce favorable outcomes [30]. Involving the lady health workers (LHWs), working under the National Program for Family Planning and Primary Health Care, in DOTS implementation and educating communities that TB is curable and treatment is possible at home free of charge can significantly improve awareness, early diagnosis and treatment adherence especially in the rural communities [35,53]. Television was the other main source of information for people in addition to health workers, consistent with the previous studies in Pakistan [31-33]. In Punjab, television coverage per household is 59.5% [37] and the current study indicated that television is the most common source of information, both in the urban and rural areas. Mass media could play a vital role in a program based on passive case finding and free diagnosis and treatment [61].

The study does have some limitations. In Pakistani culture, females are sometimes not allowed by the family heads to be interviewed by an unknown male. The data collection teams did not have female interviewers and male interviewers were not allowed to interview females in some households that resulted in the gender disproportion among those interviewed. The reason for not having female interviewers was inability of female medical students/graduates to conduct field visits due to the socio-cultural reasons in Pakistan. The health seeking behavior was intended so we cannot comment that people will actually do that.

## Conclusions and Recommendations

The study revealed poor knowledge, attitudes and practices regarding TB and the deficit was greater in the rural areas in all aspects. Therefore, area of residence, especially in countries like Pakistan where majority of the population resides in the rural areas, should be considered in tailoring communication strategies and designing future interventions for TB prevention and control.

Tuberculosis is re-emerging as a global public health problem and we need to have a better understanding of the urban and rural communities' perception of the disease to implement better prevention and control. The study highlighted the urban-rural disparities in knowledge, attitudes and practices regarding TB and its implications on TB control should not be under-estimated. On one hand there is need to train health workers and involve private practitioners while on the other hand it is necessary to health educate the masses especially those living in the rural areas. It is necessary to propagate awareness in the communities especially those living in the rural areas that they should report cough lasting longer than 3 weeks to the nearest health facility and the diagnosis and treatment of TB are free of charge. Currently, federal government manages advocacy and communication resources in all preventive programs in Pakistan; these may be de-centralized to the districts for effective advocacy and communication strategies tailored to the local needs. Population-wide health education campaigns are required to decrease stigmatization and discrimination associated with TB. Television can be recommended as a suitable medium for future campaigns provided that information should be tailored according to the needs of all people, and health workers can be involved in this regard especially in the rural areas.

## Competing interests

The authors declare that they have no competing interests.

## Authors' contributions

All authors contributed significantly in all phases of the project in accordance with uniform requirements established by International Committee of Medical Journal Editors (ICMJE) and contributors who do not meet authorship criteria are listed in the acknowledgements. All authors have read and approved the final version of manuscript.

## Supplementary Material

Additional file 1 **Map of Pakistan's Punjab province**. The file present the map of Punjab province of Pakistan with the study districts highlighted. Click here for file

Additional file 2 **Sampling, training and field management plan**. The file presents the detailed sampling plan, the trainings plan for the data collection staff, and the field management plan for the data collection activity including quality control measures. Click here for file

Additional file 3 **Survey Questionnaire**. The file presents the study questionnaire. Click here for file
