# Supplementary figures and images for: Urban-rural inequities in knowledge, attitudes and practices regarding tuberculosis in two districts of Pakistan's Punjab province
Source: Int J Equity Health. 2011 Feb 4;10:8. doi: 10.1186/1475-9276-10-8 (PMC3045313; doi:10.1186/1475-9276-10-8)

## ANNEX – III

### MAP OF PUNJAB, PAKISTAN

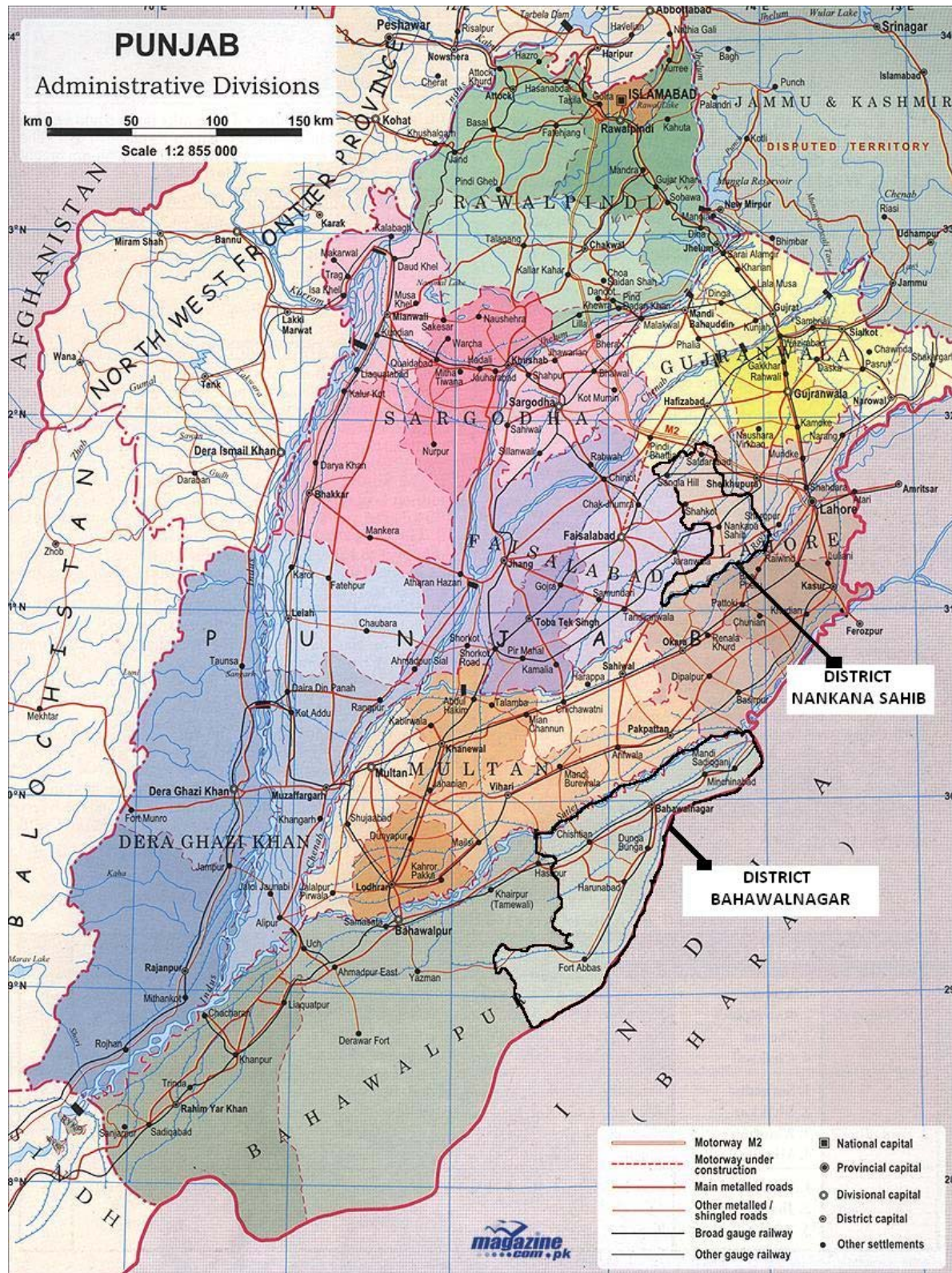

Supplement: Additional file 1 — Map of Pakistan's Punjab province. The file present the map of Punjab province of Pakistan with the study districts highlighted. [file 1475-9276-10-8-S1.PDF]
