# Supplementary material for: Urban-rural inequities in knowledge, attitudes and practices regarding tuberculosis in two districts of Pakistan's Punjab province
Source: Int J Equity Health. 2011 Feb 4;10:8. doi: 10.1186/1475-9276-10-8 (PMC3045313; doi:10.1186/1475-9276-10-8)

ANNEX – II

SAMPLING, TRAINING & FIELD MANAGEMENT PLAN

MULTI-STAGE CLUSTER SAMPLING PLAN	
**Total Sample**	**1080**
**First Stage**	
**Districts**<br>Total Districts = 2	**2**
Sample from each district	**540**
**Second Stage**	
**Zones (tehsils) from each district**<br>Total Tehsils = 6	**3**
Sample from each zone (tehsil/sub district)	**180**
**Third Stage**	
**Areas (union councils) from each zone (tehsil/sub district)**<br>5 union councils (2 urban and 3 rural) selected randomly from each tehsil. Divided into 2 areas – Area I (2 urban union councils) and Area II (3 rural union councils).<br>Total Union Councils = 30 (12 urban and 18 rural)	**5**
Sample from each Union Council	**36**
**Fourth Stage**	
**Clusters (villages/electoral wards) from each union council**<br>Total Clusters = 60	**2**
Sample from each village/electoral ward	**18**

Location Code: Location code will be written as given below:

DISTRICT NAME / ZONE (TEHSIL) NAME / AREA NUMBER / CLUSTER NUMBER

Example: Cluster 3 in Area II (Rural) of Safdarabad Zone of Nankana Sahib District will be written as **NNS / SA / AII / C3**.

CLUSTERING DETAILS

DISTRICT NANKANA SAHIB (NNS)

ZONE A – TEHSIL SHAHKOT (SH)

Areas	Clusters	Code	Sr. No.
Area I (Urban)<br>Union Council: Shahkot Urban – I<br>Union Council # SH-17	Cluster 1<br>Ward No. 3	NNS-SH-AI/C1	1-18
	Cluster 2<br>Ward No. 4	NNS-SH-AI/C2	19-36
Area I (Urban)<br>Union Council: Shahkot Urban – II<br>Union Council # SH-18	Cluster 3<br>Ward No. 13	NNS-SH-AI/C3	37-54
	Cluster 4<br>Ward No. 15	NNS-SH-AI/C4	55-72
Area II (Rural)<br>Union Council: Panwan<br>Union Council # SH-14	Cluster1<br>Panwan	NNS-SH-AII/C1	73-90
	Cluster2<br>Wali Pur Borra	NNS-SH-AII/C2	91-108
Area II (Rural)<br>Union Council: Kot Nazam Din<br>Union Council # SH-16	Cluster 3<br>Burala	NNS-SH-AII/C3	109-126
	Cluster 4<br>Mir Pur	NNS-SH-AII/C4	127-144
Area II (Rural)<br>Union Council: Karri Wala<br>Union Council # SH-20	Cluster 5<br>Karri Wala	NNS-SH-AII/C5	145-162
	Cluster 6<br>Dharo Wali	NNS-SH-AII/C6	163-180

ZONE B – TEHSIL SANGLA HILL (SG)

Areas	Clusters	Code	Sr. No.
Area I (Urban)<br>Union Council: Sangla Hill Urban – II<br>Union Council # SG-25	Cluster 1<br>Purana Chahoor	NNS-SG-AI/C1	181-198
	Cluster 2<br>Purana Shahar	NNS-SG-AI/C2	199-216
Area I (Urban)<br>Union Council: Sangla Hill Urban – III<br>Union Council # SG-26	Cluster 3<br>Islam Pura	NNS-SG-AI/C3	217-234
	Cluster 4<br>Ahmad Abad	NNS-SG-AI/C4	235-252
Area II (Rural)<br>Union Council: Bhuler, Chak No. 119/RB<br>Union Council # SG-30	Cluster 1<br>Bhuler<br>Chak No. 119/RB	NNS-SG-AII/C1	253-270
	Cluster 2<br>Marrar<br>Chak No. 45/RB	NNS-SG-AII/C2	271-288
Area II (Rural)<br>Union Council: Pandorian	Cluster 3<br>Pandorian	NNS-SG-AII/C3	289-306

Union Council # SG-31	Cluster 4<br>Sirran Wali Bhuler	NNS-SG-AII/C4	307-324
Area II (Rural)<br>Union Council: Marrar, Chak No. 42/RB<br>Union Council #	Cluster 5<br>Marrar<br>Chak No. 42/RB	NNS-SG-AII/C5	325-342
	Cluster 6<br>Kotla Kalan	NNS-SG-AII/C6	343-360
**ZONE C – TEHSIL SAFDARABAD (SA)**			
Area I (Urban)<br>Union Council: Safdarabad<br>Union Council # SA-05	Cluster 1<br>Mohalla Saeed Nagar<br>Ward No. 1	NNS-SA-AI/C1	361-378
	Cluster 2<br>Mohalla Masjid Qayyum<br>Ward No. 4	NNS-SA-AI/C2	379-396
Area I (Urban)<br>Union Council: Khanqah Dogran Urban<br>Union Council # SA-07	Cluster 3<br>Rasool Nagar<br>Ward Number 1	NNS-SA-AI/C3	397-414
	Cluster 4<br>Islam Nagar<br>Ward Number 4	NNS-SA-AI/C4	415-432
Area II (Rural)<br>Union Council: Salar Bhattian<br>Union Council # SA-01	Cluster 5<br>Salar Bhattian	NNS-SA-AII/C1	433-450
	Cluster 6<br>Mian Ali Dogran	NNS-SA-AII/C2	451-468
Area II (Rural)<br>Union Council: Nawan Pind<br>Union Council # SA-10	Cluster 3<br>Nawan Pind	NNS-SA-AII/C3	469-486
	Cluster 4<br>Adil Garh	NNS-SA-AII/C4	487-504
Area II (Rural)<br>Union Council: Abdullah Pur Kolar<br>Union Council # SA-11	Cluster 5<br>Abdullah Pur Kolar	NNS-SA-AII/C5	505-522
	Cluster 6<br>Butter-Barnala<br>Chak No. 23/RB	NNS-SA-AII/C6	523-540
**DISTRICT BAHAWALNAGAR (BWN)**			
**ZONE A – TEHSIL BAHAWALNAGAR (BN)**			
Areas	***Clusters***	***Code***	***Serial No***
Area I (Urban)<br>Union Council: Jinnah Colony<br>Union Council # 3	Cluster 1<br>Jinnah Colony	BWN-BN-AI/C1	540-558
	Cluster 2<br>Madni Colony	BWN-BN-AI/C2	559-576

Area I (Urban)<br>Union Council: Nazam Pura<br>Union Council # 4	Cluster 3<br>Nazam Pura	BWN-BN-AI/C3	577-594
	Cluster 4<br>Mohalla Kolho Wala	BWN-BN-AI/C4	595-612
Area II (Rural)<br>Union Council: Moosa Bhutta<br>Union Council # 15	Cluster 1<br>Mari Mian Sahib	BWN-BN-AII/C1	613-630
	Cluster 2<br>Meeran Shah	BWN-BN-AII/C2	631-648
Area II (Rural)<br>Union Council: Soondha<br>Union Council # 28	Cluster 3<br>Chak Soondha	BWN-BN-AII/C3	649-666
	Cluster 4<br>Chak Gulab Ali	BWN-BN-AII/C4	667-684
Area II (Rural)<br>Union Council: Mohar Wali<br>Union Council # 22	Cluster 5<br>Mohar Wali	BWN-BN-AII/C5	685-702
	Cluster 6<br>Basti Jagar Wali	BWN-BN-AII/C6	703-720
**ZONE B – TEHSIL CHISHTIAN (CH)**			
Area I (Urban)<br>Union Council: Chishtian Urban<br>Union Council # 36	Cluster 1:<br>Baldia Colony	BWN-CH-AI/C1	721-738
	Cluster 2:<br>Muslim Town	BWN-CH-AI/C2	739-756
Area I (Urban)<br>Union Council: Chishtian Urban<br>Union Council # 33/2	Cluster 3:<br>Satellite Town	BWN-CH-AI/C3	757-774
	Cluster 4:<br>Noor Pura	BWN-CH-AI/C4	775-792
Area II (Rural)<br>Union Council: Chak No. 42/F<br>Union Council # 36	Cluster 1:<br>Chak No. 102/F	BWN-CH-AII/C1	793-810
	Cluster 2:<br>Chak No. 23/G	BWN-CH-AII/C2	811-828
Area II (Rural)<br>Union Council: Chak No. 52/F<br>Union Council # 45	Cluster 3:<br>Chak No. 54/F	BWN-CH-AII/C3	829-846
	Cluster 4:<br>Bakhshan Khan<br>Chak No. 9/FW	BWN-CH-AII/C4	847-864
Area II (Rural)<br>Union Council: Chak No. 98/F<br>Union Council # 43	Cluster 5:<br>Chak No. 134 - Murad	BWN-CH-AII/C5	865-882
	Cluster 6:<br>Chak No. 132- Murad	BWN-CH-AII/C6	883-900
**ZONE C – TEHSIL HAROONABAD (HA)**			
Area I (Urban)<br>Union Council: Baldia Colony<br>Union Council # 80	Cluster 1<br>Ward No. 5	BWN-HA-AI/C1	900-918
	Cluster 2<br>Ward No. 6	BWN-HA-AI/C2	919-936

Area I (Urban)<br>Union Council: Madina Colony<br>Union Council # 78	Cluster 3<br>Ward No. 8	BWN-HA-AI/C3	937-954
	Cluster 4<br>Ward No. 9	BWN-HA-AI/C4	955-972
Area II (Rural)<br>Union Council: Chak No. 58/4R<br>Union Council # 77	Cluster 1<br>Chak No. 85/6R	BWN-HA-AII/C1	973-990
	Cluster 2<br>Chak No. 70/4R	BWN-HA-AII/C2	991-1008
Area II (Rural)<br>Union Council: Chak No. 101/6R<br>Union Council # 92	Cluster 3<br>Chak No. 68/4R	BWN-HA-AII/C3	1009-1026
	Cluster 4<br>Chak No. 69/4R	BWN-HA-AII/C4	1027-1044
Area II (Rural)<br>Union Council: Chak No. 105/6R<br>Union Council # 96	Cluster 5<br>Chak No. 61/4R	BWN-HA-AII/C5	1045-1062
	Cluster 6<br>Chak No. 62/4R	BWN-HA-AII/C6	1063-1080

TRAINING PLAN

TRAINING OF TRAINERS		
**Facilitators**	Principal Investigator and Co Investigators	2
**Participants**	Zonal Supervisors & Area In-charges	18
**Venue**	District Headquarters	

TRAINING OF DATA COLLECTORS		
**DISTRICT NANKANA SAHIB**		
**Zone A - Nankana Sahib**		
**Facilitators**	2 Area In-charges trained at TOT, Zonal Supervisor and Co Investigator	4
**Participants**	Data Collectors	10
**Venue**	District Headquarter	
**Zone B - Shahkot**		
**Facilitators**	2 Area In-charges trained at TOT, Zonal Supervisor and Co Investigator	4
**Participants**	Data Collectors	10
**Venue**	Rural Health Centre	
**Zone C - Safdarabad**		
**Facilitators**	2 Area In-charges trained at TOT, Zonal Supervisor and Co Investigator	4
**Participants**	Data Collectors	10
**Venue**	Rural Health Centre	
**DISTRICT BAHAWALNAGAR**		
**Zone A - Bahawalnagar**		
**Facilitators**	2 Area In-charges trained at TOT, Zonal Supervisor and Co Investigator	4
**Participants**	Data Collectors	10
**Venue**	District Headquarter	
**Zone B - Chishtian**		
**Facilitators**	2 Area In-charges trained at TOT, Zonal Supervisor and Co Investigator	4
**Participants**	Data Collectors	10
**Venue**	Tehsil Headquarters Hospital	
**Zone C - Haroonabad**		
**Facilitators**	2 Area In-charges trained at TOT, Zonal Supervisor and Co Investigator	4
**Participants**	Data Collectors	10
**Venue**	Tehsil Headquarters Hospital	

FIELD MANAGEMENT PLAN

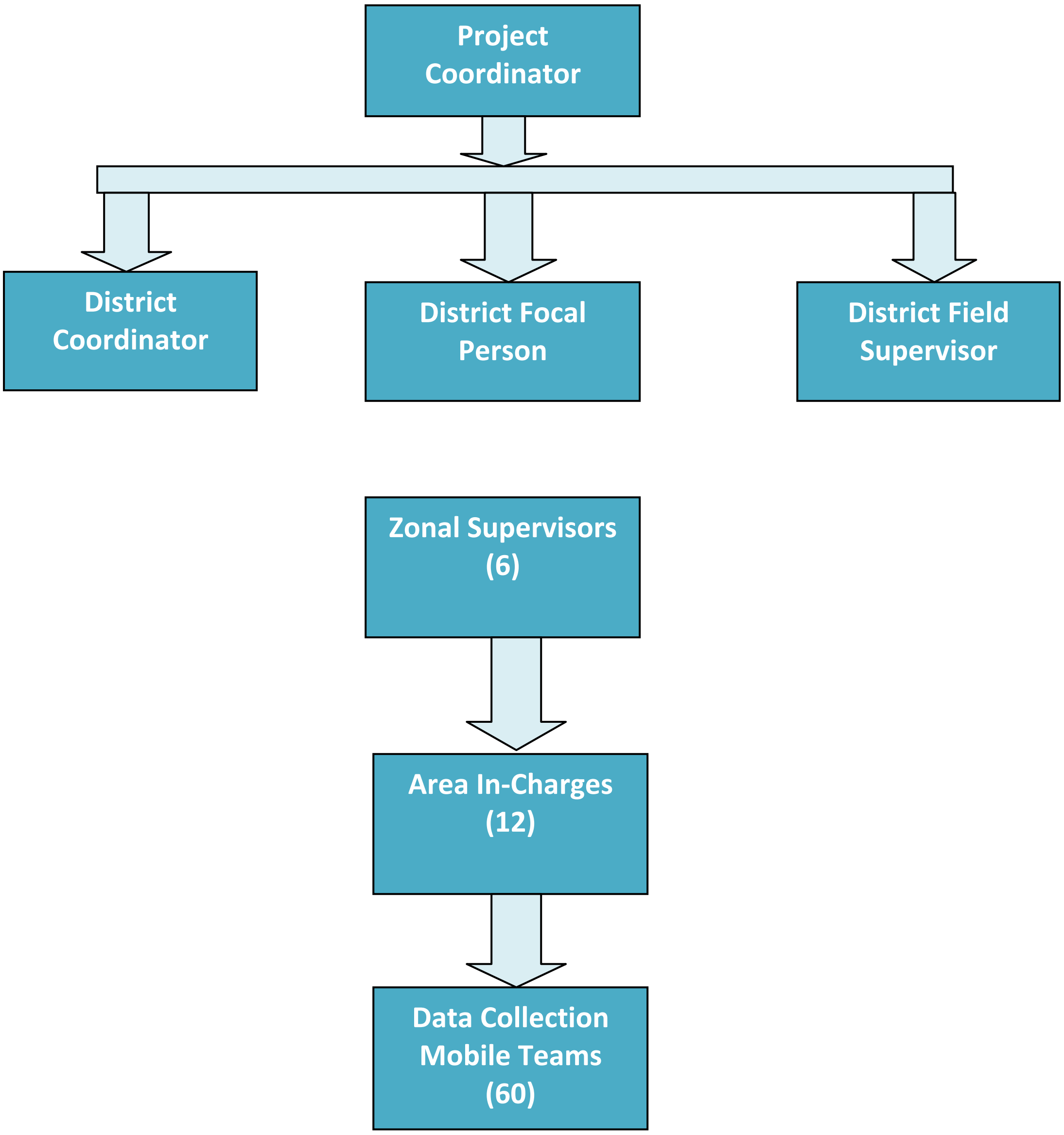

Supplement: Additional file 2 — Sampling, training and field management plan. The file presents the detailed sampling plan, the trainings plan for the data collection staff, and the field management plan for the data collection activity including quality control measures. [file 1475-9276-10-8-S2.PDF]
