# Supplementary material for: Urban-rural inequities in knowledge, attitudes and practices regarding tuberculosis in two districts of Pakistan's Punjab province
Source: Int J Equity Health. 2011 Feb 4;10:8. doi: 10.1186/1475-9276-10-8 (PMC3045313; doi:10.1186/1475-9276-10-8)
Supplement: Additional file 3 — Survey Questionnaire. The file presents the study questionnaire. [file 1475-9276-10-8-S3.PDF]

ANNEX – I

SURVEY QUESTIONNAIRE

Information to be read to the respondent:

We are from Department of Health, District ______ and we are working on a project concerned with TB control. We wish to learn about your knowledge, attitudes and practices regarding tuberculosis. We hope to understand your needs and the best way to bring information to you, as well as barriers to seeking medical care. The information you provide will be used to improve TB control. Your answers will not be released to anyone and will remain anonymous. Your name will not be written on the questionnaire or be kept in any other records. Your participation is voluntary and you may choose to stop the interview at any time. The interview will take 15 minutes, may I start now?

Thank you for your assistance. (*If permission is given start the interview*)

Date (dd/mm/yy): ___ / ___ / ___	Household Number: __________	Location Code: ____ / ___ / ___

Interviewer Name and designation: ______________________________	Area: ☐ Urban ☐ Rural
Zone Name: ....<br>Area Number:	Zonal Supervisor: ....<br>Area In-Charge: ....
Union Council:<br>Name...<br>Number..	Cluster (Village/Ward):<br>Name ....<br>Number ....

Interviewer Instructions: *Place an (**X**) in the box of the selected answer(s). Do not read responses unless the directions indicate. Instructions are given in italics.*

General, Demographic and Socioeconomic Questions

1. Age?
1. ☐ Under 30
2. ☐ 31–40
3. ☐ 41–50
4. ☐ Over 50

2. Sex?
1. ☐ Male
2. ☐ Female

3. What is the highest level of education you have completed?
1. ☐ No school
2. ☐ Primary
3. ☐ High school
4. ☐ College
5. ☐ Higher education (professional or post-graduate)
6. ☐ Religious schooling only
7. ☐ Other (*please specify*) ____

4. Total family members?

____

5. Working family members?

____

6. Total monthly income of the family (PKR)?

____

7. Condition of house?
1. ☐ Muddy
2. ☐ Cemented
3. ☐ Mixed

TB Knowledge

8. What are the signs and symptoms of TB? *(Please check all that are mentioned)*
1. ☐ Cough
2. ☐ Cough that lasts longer than 3 weeks
3. ☐ Coughing up blood
4. ☐ Severe headache
5. ☐ Nausea
6. ☐ Weight loss
7. ☐ Fever
8. ☐ Fever without clear cause that lasts more than 7 days
9. ☐ Chest pain
10 ☐ Shortness of breath
11 ☐ Ongoing fatigue
12 ☐ Do not know
13 ☐ Other *(please specify)*: ____

9. How can a person get TB? *(Please check all that are mentioned)*
1. ☐ Through handshakes
2. ☐ Through the air when a person with TB coughs or sneezes
3. ☐ Through sharing dishes
4. ☐ Through eating from the same plate
5. ☐ Through touching items in public places (doorknobs, handles in transportation)
6. ☐ Do not know
7. ☐ Other (*please explain*): ____

10. How can a person prevent getting TB? *(Please check all that are mentioned)*
1. ☐ Avoid shaking hands
2 ☐ Covering mouth and nose when coughing or sneezing
3. ☐ Avoid sharing dishes
4. ☐ Washing hands after touching items in public places
5. ☐ Closing windows at home
6. ☐ Through good nutrition
7. ☐ By praying
8. ☐ By drugs
9. ☐ By vaccination (BCG)
10 ☐ Do not know
11 ☐ Other *(please explain)*: ____

11. In your opinion, who can be infected with TB? (*Please check all that are mentioned)*
1. ☐ Anybody
2. ☐ Only poor people
3. ☐ Only homeless people
4. ☐ Only alcoholics
5. ☐ Only drug users
6. ☐ Only people living with HIV/AIDS
7. ☐ Only people who have been in prison
8. ☐ Other (*please explain*): ____

12. How can someone with TB be cured? *(Check all that are mentioned)*
1. ☐ Herbal remedies
2. ☐ Home rest without medicine
3. ☐ Praying
4. ☐ Specific drugs given by health center
5. ☐ DOTS
6. ☐ Do not know
7. ☐ Other *(please specify)*: ____

13. Where can TB be cured?
1. ☐ Private clinic
2. ☐ Government clinics or hospital
3. ☐ Traditional or homeopathic healer
4. ☐ Clinic run by a nongovernmental organization or church
5. ☐ Other *(please specify)*: ____

14. How long does TB treatment last?
1. ☐ Less than 1 month
2. ☐ 1 to 3 months
3. ☐ 3 to 6 months
4. ☐ 6 to 9 months
5. ☐ More than 9

15. How expensive do you think TB diagnosis and treatment is in this country? *(Please check one)*
1. ☐ It is free of charge
2. ☐ It is reasonably priced
3. ☐ It is somewhat/moderately expensive

4. ☐ It is very expensive

If respondent gives monetary amount, note the amount here: _________________

16. In your opinion, how serious a disease is TB? *(Check one)*
1. ☐ Very serious
2. ☐ Somewhat serious
3. ☐ Not very serious

17. How serious a problem do you think TB is in your country/region? *(Check one)*
1. ☐ Very serious
2. ☐ Somewhat serious
3. ☐ Not very serious

TB Attitudes and Practices

18. Who would you talk to about your illness if you had TB?
(Check all that are mentioned)
1. ☐ Doctor or other medical worker
2. ☐ Spouse
3. ☐ Parent
4. ☐ Child(ren)
5. ☐ Other family member
6. ☐ Close friend
7. ☐ No one
8. ☐ Other *(please specify)*: _______________________________________

19. What would you do if you thought you had symptoms of TB? (Check all that apply.)
1. ☐ Go to health facility
2. ☐ Go to pharmacy
3. ☐ Got to traditional healer
4. ☐ Pursue other self-treatment options (herbs, etc.)
5. ☐ Other *(please specify)*: _______________________________________

20. If you had symptoms of TB, when would you go to the health facility? *(Please check one)*
1. ☐ When treatment on my own does not work
2. ☐ When symptoms that look like TB signs last for 3–4 weeks
3. ☐ As soon as I realize that my symptoms might be related to TB
4. ☐ I would not go to the doctor

21. In your community, how a person, who has TB, is usually regarded/treated?
1. ☐ Most people reject him or her
2. ☐ Most people are friendly, but they generally try to avoid him or her
3. ☐ The community mostly supports and helps him or her
4. ☐ Other *(please explain)*: _______________________________________

22. How do you feel towards people who have TB?
1. ☐ Sympathy
2. ☐ Hate
3. ☐ Friendly but I will try to avoid him or her
4. ☐ I will support and help him or her

5. ☐ Others *(please explain)*: ____

23. What would be the quality of life of person with TB?

1. ☐ Normal
2. ☐ Poor
3. ☐ Very Poor
4. ☐ Good

Sources of information

24. Is information of TB available to you?

1. ☐ Yes
2. ☐ No

25. Where do you currently get information about TB?

1. ☐ TV
2. ☐ Radio
3. ☐ Newspapers and magazines
4. ☐ Billboards
5. ☐ Brochures, posters and other printed materials
6. ☐ Health workers
7. ☐ Family, friends, neighbors and colleagues
8. ☐ Religious leaders
9. ☐ Teachers
10. ☐ Other (*please explain*): ____

26. What hours (PST)?

1. ☐ Morning (6 am –10 am)
2. ☐ Noon (10am-2pm)
3. ☐ After noon (2pm-6pm)
4. ☐ Evening (6pm-8pm)
5. ☐ Night (8pm onwards)

27. What channels? (*Please specify*) ____

Thank you very much for participating in our survey.
